# Effect of TLR4/MyD88 Signaling Pathway on Expression of IL-1β and TNF-α in Synovial Fibroblasts from Temporomandibular Joint Exposed to Lipopolysaccharide

**DOI:** 10.1155/2015/329405

**Published:** 2015-02-24

**Authors:** Xuefen Lin, Jingjing Kong, Qingting Wu, Yingying Yang, Ping Ji

**Affiliations:** ^1^Stomatological Hospital of Shandong University, Number 44, Wen Hua Xi Lu, Shandong Province, Jinan 250012, China; ^2^Key Laboratory of Oral Biomedicine of Shandong Province, Number 44, Wen Hua Xi Lu, Shandong Province, Jinan 250012, China

## Abstract

Accumulating evidence from previous studies suggested that interleukin-1 (IL-1*β*) and tumor necrosis factor-*α* (TNF-*α*) play an important role in pathogenesis of temporomandibular disorders (TMD). However, the cell surface receptors and the intracellular signal pathways leading to these cytokines expression are not fully understood. In the current study, we investigated the roles of Toll-like receptor 4 (TLR4) and its adaptor myeloid differentiation factor 88 (MyD88) in the expression of IL-1*β* and TNF-*α* in synovial fibroblasts (SFs) separated from rat temporomandibular joint (TMJ) with lipopolysaccharide (LPS) stimulation. The results showed that treatment with LPS could increase TLR4, MyD88, IL-1*β*, and TNF-*α* expression at both mRNA and protein levels. In addition, increased expression of IL-1*β* and TNF-*α* could be blocked by treatment with TAK-242, a blocker of TLR4 signaling, and also by MyD88 inhibitory peptide (MIP). These findings suggested that maybe TLR4/MyD88 signal transduction pathway participates in enhanced expression of IL-1 and TNF-*α* in patients with TMD. The activation of TLR4/MyD88 signal transduction pathway which results in production of proinflammatory factors may play a role in the pathogenesis of TMD.

## 1. Introduction

Temporomandibular disorder (TMD) is comprised of a group of symptoms like pain and clicking in Temporomandibular Joint (TMJ), dysfunction associated with pain in the muscles of mastication, and limited mouth opening. A large number of scholars have conducted various studies in its aetiology. The initiation of TMD is considered to involve many risk factors, such as occlusal interferences [1], psychological factors [2], and biomechanical and neuromuscular factors [3]. Nevertheless, there are no definitive conclusions about the mechanisms of the initiation and development of this disease. Recently, there has been robust interest in the inflammatory response of TMD. Various inflammatory mediators are thought to be involved in its pathophysiology, including proinflammatory cytokines [4, 5] and matrix metalloproteinases (MMPs) [6, 7]. Among them, interleukin- (IL-) 1*β* and tumor necrosis factor- (TNF-) *α* appear to play an important role in the synovium and cartilage damage. IL-1*β* is induced as an inactive promolecule (pro-IL-1*β*) by immune cells and then cleaved into the active form (mature IL-1*β*) by caspase-1 [8, 9] and subsequently secreted. The IL-1*β* and TNF-*α* levels in synovial fluids of TMJ with TMD are detected [10–12]. The elevated concentrations of IL-1*β* and TNF-*α* are associated with TMJ pain and joint destruction [13, 14]. Additionally, the cellular sources of enhanced IL-1*β* and TNF-*α* in synovial fluids of TMD were suggested to be mainly synovial lining cells and endothelial cells of blood vessels [15]. However, the cell surface receptors which help ligand recognition and binding and the intracellular signal transduction pathways leading to cytokines expression are not thoroughly understood.

TLR4 is a member of the TLR (Toll-like receptor) family of transmembrane proteins recognize conserved pathogen-associated molecular patterns like lipopolysaccharide (LPS), viral double-stranded RNA, bacterial flagella, and viral and bacterial CpG DNA and generate innate immune responses to pathogens by activating a cascade of proinflammatory events [16]. Recent studies have found that endogenous ligands such as saturated free fatty acids [17] and high mobility group box-1 protein [18] can also activate TLR4. When a ligand binds to TLR4 and its coreceptors CD14 and MD-2, the adaptor molecules Toll/IL-1 receptor (TIR) domain-containing adaptor protein (TIRAP), myeloid differentiation factor 88 (MyD88), TIR domain-containing adapter-inducing IFN-*β* (TRIF), and TRIF-related adaptor molecule (TRAM) are recruited to the TIR domain of TLR4. This protein-protein interaction cascade enables downstream signalling and mediates activation of a transcriptional factor and nuclear factor (NF)-*κ*B, resulting in induction of proinflammatory genes, such as those encoding TNF-*α*, IL-6 and IL-1*β* [19, 20].

Previous studies have demonstrated that the TLR4 signaling pathways play an important role in the progression of many diseases by mediating the expression of proinflammatory cytokines. Edfeldt et al. suggested that hyporesponsive TLR4 polymorphisms affect the susceptibility to myocardial infarction in men and that TLR4-mediated innate immunity plays a role in the pathogenesis of myocardial infarction [21]. A report identified that the interaction TLR4 signaling pathway (including MyD88, TRIF, ASK1, and p38) is involved in the development of Lung ischemia reperfusion injury (LIRI) [22]. Kim et al. cultivated the cartilage cells isolated from patients with osteoarthritis and detected increased expression of TLR4 mRNA [23]. However, not much is known about the correlations between TLR4 signaling and the pathogenesis of TMD.

In the present study, we describe the change of TLR4, MyD88, IL-1*β*, and TNF-*α* expression under the LPS stimulation in synovial fibroblasts (SFs) from TMJ. Besides, we use a specific inhibitor (TAK-242) to investigate whether TLR4 is involved in the expression of IL-1*β* and TNF-*α* with LPS stimulation. Next, MyD88 inhibitory peptide (MIP) was used to determine whether the effects are dependent at least in part upon MyD88.

## 2. Materials and Methods

### 2.1. Isolation and Culture of SFs

Five male wistar rats (6-week-old, obtained from the Shandong University Center of Laboratory Animals, China) were used as a source of SFs. Rats were submitted to euthanasia in a CO_2_ chamber, and synovial tissue was harvested from the TMJ according to a described procedure with minor modifications [24]. The protocol was approved by the Animal Care and Use Committee at the Shandong University. Briefly, the samples were washed extensively with phosphate buffered saline (PBS) and then minced into 1 mm^3^ pieces and plated onto tissue culture dishes with a medium consisting of Dulbecco's modified Eagle medium (DMEM, Gibco, NY, USA) supplemented with 5 mM HEPES buffer, 100 U/mL of penicillin G (Sigmae-Aldrich, St. Louis, MO), 50 mg/mL of gentamicin (Sigma-Aldrich, St. Louis, MO), and 15% heat-inactivated fetal bovine serum (FBS, Gibco, NY, USA) at 37°C in an atmosphere of 5% CO_2_, 5% O_2_, and 90% N_2_. When cells grown from the explants reached confluency, all SFs from different donor animals were trypsinized and subcultured together in DMEM supplemented with 10% FBS and antibiotics. Near confluent cells between passage 3 and 6 were used for experiments.

### 2.2. Experiment Protocol

Isolated SFs were seeded at a density of 1 × 10^5^ cells/cm^2^ in 6-well or 24-well plates and incubated overnight in DMEM with 10% FBS. Prior to the treatment, the serum-containing medium of SFs was removed and serum-free medium was added. After synchronization in the serum-free culture medium for 16 h, the regular medium was replaced and SFs were treated with LPS, TAK-242, and MIP.

For LPS treatment, each culture plate was treated by LPS (*Escherichia coli*, strain 0128:B12, Sigma) for 1, 6, 12, and 24 h, at final concentrations of 100 ng/mL. Control cultures containing no LPS were grown in parallel. On the other hand, SFs were incubated in the presence of increasing concentrations of LPS (0.1, 1, 10, and 100 ng/mL) for 12 h. Control cultures containing no LPS were grown in parallel.

To evaluate the effect of TLR4 on the expression of IL-1*β* and TNF-*α* in SFs, the cells were pretreated with 1 *μ*M TAK-242 (Invitrogen, San Diego, CA, USA) for 2 h before LPS (100 ng/mL) stimulation for 6 h or 12 h. The inhibitor was reconstituted in dimethyl sulphoxide (DMSO), and control cells were preincubated with equivalent amounts of 0.001% DMSO alone.

To evaluate the effect of MyD88 on the expression of IL-1*β* and TNF-*α* in SFs, the cells were pretreated with 100 *μ*M MIP (Imgenex, San Diego, CA) for 2 h before LPS (100 ng/mL) stimulation for 6 h or 12 h, and control cells were preincubated with peptide lacking the MyD88 binding domain (Imgenex).

### 2.3. Immunofluorescence

The morphology of SFs was confirmed by immunofluorescence staining detected marker proteins CD68 and vimentin (macrophage and fibroblasts markers, resp.) [25]. SFs were washed with PBS three times and fixed with 4% paraformaldehyde. After permeabilizing cell membranes with ice cold 0.3% Triton X-100 for 10 min, incubating them for 1 h in blocking solution containing PBS, 0.5% (m/v) BSA. Cells were incubated with CD68 and vimentin antibodies (1 : 500, Cell Signaling, Beverly, MA, USA) respectively, followed by incubation with FITC-conjugated goat anti-rabbit secondary antibody (Zhongshan, Beijing, China) for 30 min. Finally, cells were counterstained with DAPI (Zhongshan, Beijing, China) and visualized by immunofluorescence microscopy (Olympus CX-RFL-2, Tokyo, Japan).

### 2.4. Real-Time Quantitative PCR

Total RNA was extracted from cells using Trizol (Invitrogen, Carlsbad, CA, USA) according to the manufacturer's protocol. The first strand complementary DNA (cDNA) was synthesized by reverse transcription using SYBR Prime Script TM RT reagent Kit (Takara, Dalian, China). The levels of target mRNA in cells were analyzed by quantitative real-time PCR using SYBR Green I dye (Takara, Dalian, China). The primer pairs used for PCR were as follows: forward 5′-CCCAGGCAGTCAGATCATCTTCT-3′ and reverse 5′-ATGAGGTAC AGGCCCTCTGAT-3′ for TNF-*α*, forward 5′-CCTGTGCAATTTGACCATTG-3′ and reverse 5′- AAGCATTCCCACCTT TGTTG-3′ for TLR4, forward 5′-TGCAGAGCA AGGAATGTGAC-3′ and reverse 5′-AGGATGCTGGGGAACTCTTT-3′ for MyD88,, forward 5′-ACAAGGAGAGACAAGCAACGA-3′ and reverse 5′-TCTGCTTGAGAGGTGCTGATG-3′ for IL-1*β*, and forward 5′-GAAGGTGAAGGTCGGAGTCG-3′ and reverse 5′-GAAGATGGTGATGGGATTTC-3′ for glyceraldehyde-3-phosphate dehydrogenase (GAPDH).

The amplifications were performed in triplicate on a LightCycler 480 QPCR System (Roche Diagnostics Ltd., Bern, Switzerland). Each gene was normalized against the corresponding GAPDH levels and relative gene expression of each sample was fold change (2^−ΔΔCt^) using the control group as calibrator.

### 2.5. Western Blot

For total cellular protein, cells were collected and lysed with lysis buffer containing 20 mM Tris-HCl (pH7.5), 1% Triton X-100, 150 mM NaCl, 2.5 mM EDTA, and 1 mM PMSF. The lysates were centrifuged at 10,000 rpm for 30 min (Kubota 6930, Tokyo, Japan) to remove unbroken cells, nuclei, and other organelles. The supernatant containing plasma membrane was recovered and stored at −70°C for analysis. Equal amounts of proteins (5 *μ*g) were electrophoresed in 10% sodium dodecyl sulfate-polyacrylamide gel electrophoresis gels (SDS-PAGE) and transferred onto a polyvinylidene difluoride (PVDF) membrane (Bio-Rad, Hercules, CA, USA). The membrane was blocked in fresh 5% dry nonfat milk in Tris-buffered saline/0.05%Tween-20 (TBST) for 1 h, and then incubated with the primary antibodies against TLR4, MyD88, pro-IL-1*β*, TNF-*α* (1 : 1000, Cell Signaling, Beverly, MA, USA) and GAPDH (Santa Cruz Biotechnology, Santa Cruz, CA, USA), respectively, overnight at 4°C. Then the membranes were incubated with horseradish peroxidase (HRP)-conjugated secondary anti-rabbit antiserum (Santa Cruz, CA, USA, 1 : 5000 dilution in TBS) for 1 h. Immunoreactive proteins were visualized with an enhanced chemiluminescence (ECL) and captured on an X-ray film. Protein levels were quantified using Image J software (National Institutes of Health, CA, USA).

### 2.6. ELISA

The cell-free culture medium from each sample was collected, centrifuged, and stored at −80°C until tested. Mature IL-1*β* and TNF-*α* proteins secreted into the cell culture media were measured using the commercial ELISA kits (Uscn, Hubei, China) following the manufacturer's instructions. The protein levels of mature IL-1*β* and TNF-*α* were expressed as pg/mL in cell culture media.

### 2.7. Statistical Analysis

Normally distributed variables were expressed as means ± SD. Unpaired Student's *t*-test or ANOVA were used to compare differences between groups. Differences in data values were defined significant at a *P* < 0.05 using SPSS statistical software package Version 17.0.

## 3. Results 

### 3.1. Characterisation of SFs by Immunofluorescence

**Figure 1:**
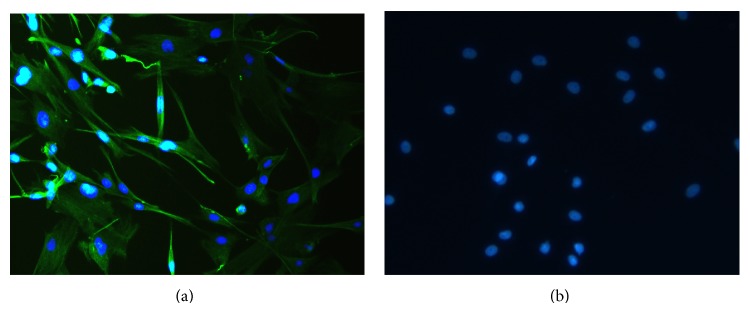
Characterisation of SFs by immunofluorescence. (a) SFs from the third to sixth passage were stained with antibody against vimentin. (b) SFS from the third to sixth passage were stained with antibody against CD68.

To test whether the cells used in these experiments were homogeneous with respect to fibroblast-like cells, cells from the third to sixth passage were characterised by immunofluorescence. It was shown that all of cells were positively stained by vimentin antibody (Figure 1(a)), while negatively stained by CD68 antibody (Figure 1(b)). The results suggested that the cells are mesodermal in origin and indicate features of fibroblasts.

### 3.2. Expression of IL-1*β* and TNF-*α* in SFs in Response to LPS

**Figure 2:**
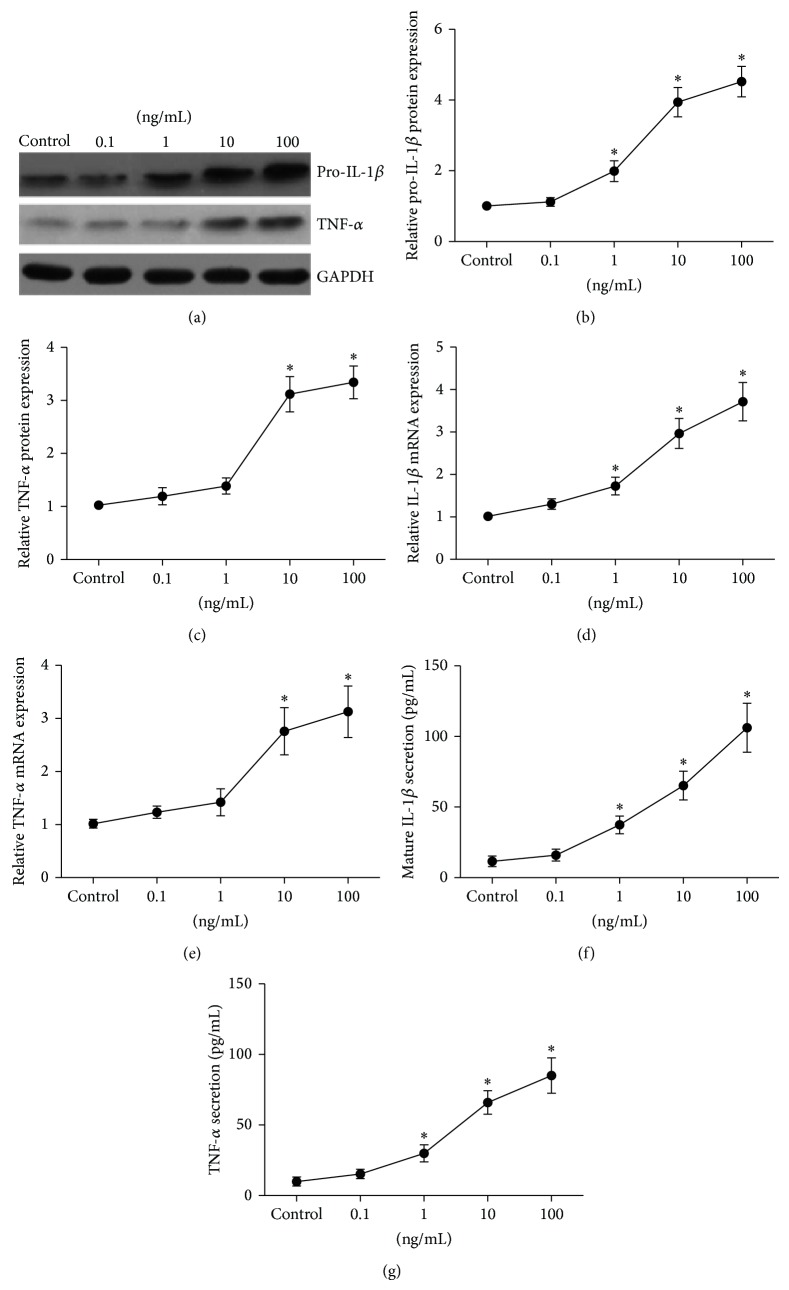
LPS induced IL-1*β* and TNF-*α* expression in SFs in a dose-dependent manner. SFs were treated with LPS (0.1, 1, 10, 100 ng/mL) for 12 h, cellular lysates were subjected to Western blotting to detect the protein expression of pro-IL-1*β* and TNF-*α* (a), and the relative expression of pro-IL-1*β* (b) and TNF-*α* (c) was calculated. The expression of IL-1*β* (d) and TNF-*α* (e) mRNA was determined by real time quantitative PCR. Mature IL-1*β* (f) and TNF-*α* (g) proteins secreted into the cell culture media were measured by ELISA. Data shows all the values from independent samples of *n* = 3, ^*^ *P* < 0.05 versus nonstimulated control.

In the current study, we mainly investigated the change of proinflammatory mediators expression under the LPS stimulation in SFs. As seen in Figures 2(b), and 2(c), the results showed a dose-dependent increase of pro-IL-1*β* and TNF-*α* proteins expression in SFs with treated by LPS (0.1, 1, 10, 100 ng/mL) for 12 h, with the peak expression at 100 ng/mL of the stimulation. Consistent with their proteins increasing, their mRNAs were also elevated, maximized at 100 ng/mL of the stimulation (Figures 2(d), and 2(e)). Also, LPS enhanced TNF-*α* and mature IL-1*β* protein levels secreted to the cell culture media in a dose-dependent manner (Figures 2(f), and 2(g)).

**Figure 3:**
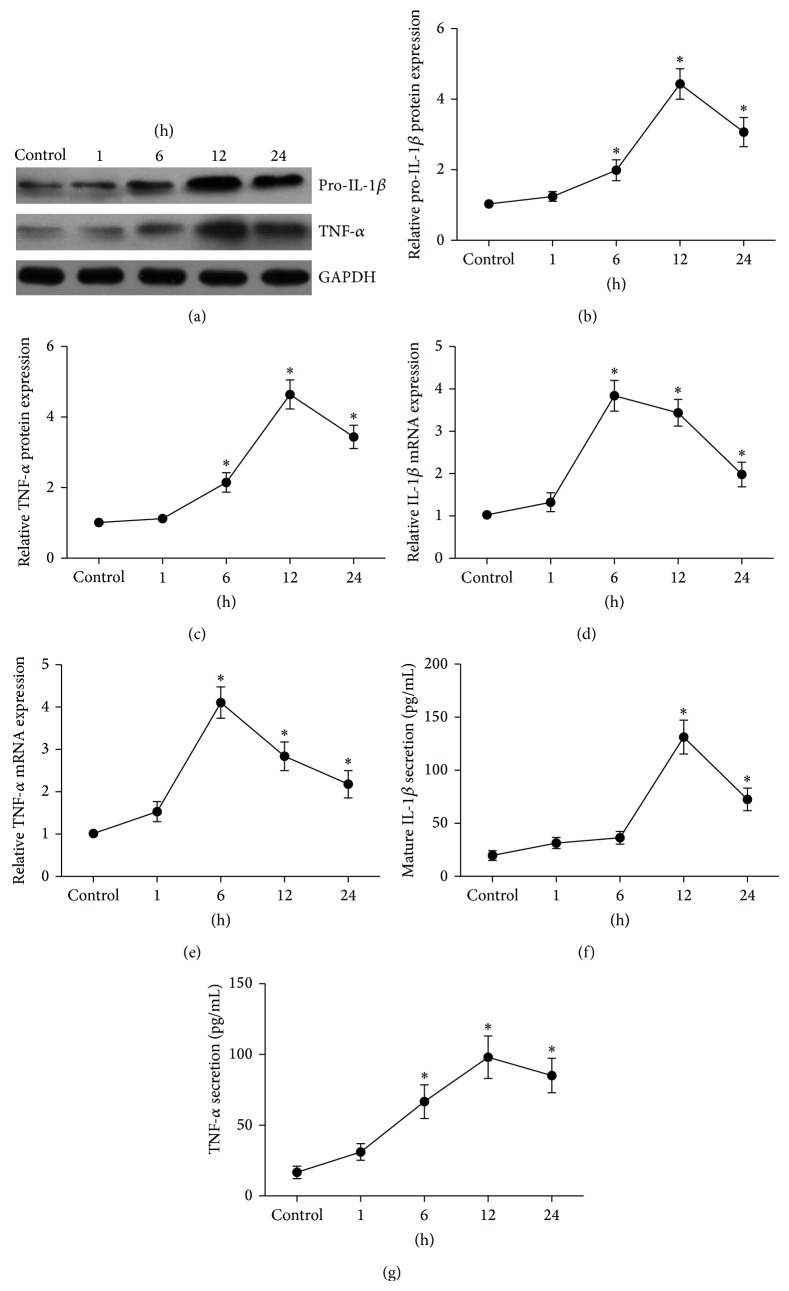
LPS induced IL-1*β* and TNF-*α* expression in SFs in a time-dependent manner. SFs were treated with LPS (100 ng/mL) for 1, 6, 12, 24 h, cellular lysates were subjected to Western blotting to detect the protein expression of pro-IL-1*β* and TNF-*α* (a), and the relative expression of pro-IL-1*β* (b) and TNF-*α* (c) was calculated. The expression of IL-1*β* (d) and TNF-*α* (e) mRNA was determined by real time quantitative PCR. Mature IL-1*β* (f) and TNF-*α* (g) proteins secreted into the cell culture media were measured by ELISA. Data shows all the values from independent samples of *n* = 3, ^*^ *P* < 0.05 versus nonstimulated control.

Next, SFs were treated with LPS (100 ng/mL) for 1, 2, 6, 12, 24 h. The proteins expression of pro-IL-1*β* and TNF-*α* showed a time-course dependent increase, with the peak expression at 12 h of the stimulation (Figures 3(b), and 3(c)). Consistent with their proteins increasing, their mRNA expresssion were also enhanced, with the peak expression at 6 h of the stimulation (Figures 3(d), and 3(e)). Also, LPS increased TNF-*α* and mature IL-1*β* protein levels secreted to the cell culture media in a time-dependent manner (Figures 3(f), and 3(g)).

### 3.3. LPS Stimulates TLR4 and MyD88 Expression in SFs

**Figure 4:**
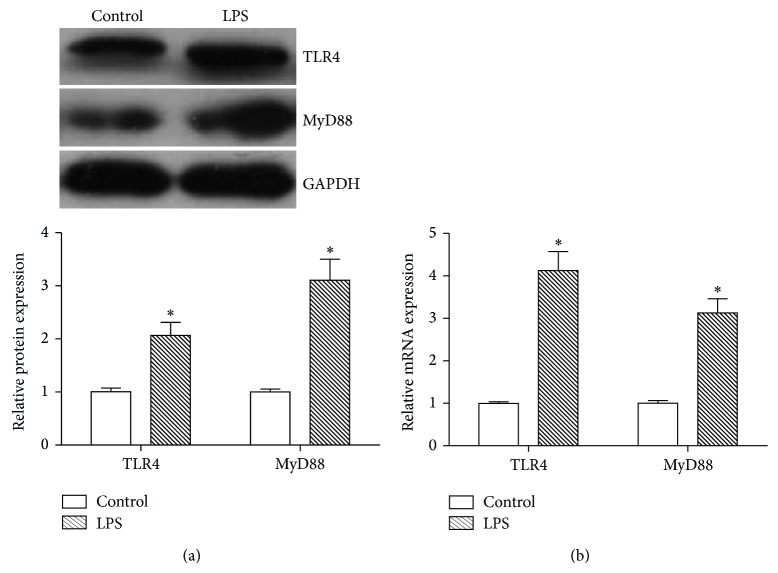
LPS induced TLR4 and MyD88 expression in SFs. SFs were treated with LPS (100 ng/mL) for 6 h or 12 h. Cell lysates were collected (at 12 h) for analyzing the protein expression of TLR4 and MyD88 (a) by Western blot. Total RNAs were extracted (at 6 h) for detecting mRNAs of TLR4 and MyD88 (b) by real-time quantitative PCR. Data shows all the values from independent samples of *n* = 3, ^*^ *P* < 0.05 versus nonstimulated control.

In this study, SFs were treated with LPS (100 ng/mL) for 6 h or 12 h. Cell lysates were collected (at 12 h) for analyzing the protein expression of TLR4 and MyD88, and RNAs were extracted (at 6 h) for detecting mRNAs. As shown in Figure 4, LPS could enhance not only TLR4 but also MyD88 expression at both protein (Figure 4(a)) and mRNA (Figure 4(b)) levels in SFs compared to untreated cells.

### 3.4. Effect of TLR4 on the Expression of IL-1*β* and TNF-*α* in SFs

**Figure 5:**
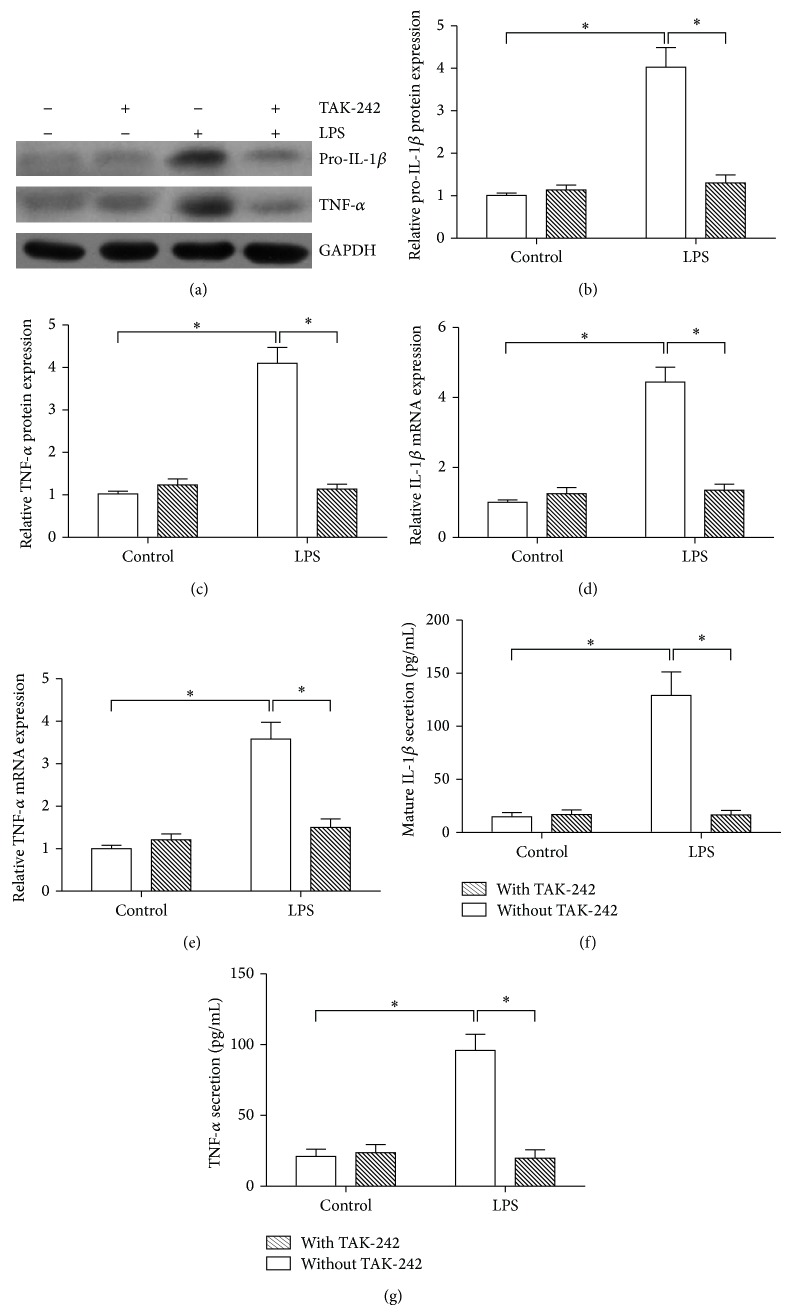
Effect of TLR4 on the expression of IL-1*β* and TNF-*α* in SFs. SFs were pre-treatment with or without TAK-242, then stimulated with LPS. Cell lysates were collected (at 12 h) for analyzing the protein expression by Western blot (a), and the relative expression of pro-IL-1*β* (b) and TNF-*α* (c) was calculated. Total RNAs were extracted (at 6 h) for detecting mRNAs of IL-1*β* (d) and TNF-*α* (e) by real-time quantitative PCR. The cell-free culture medium from each sample was collected at 12 h, and the mature IL-1*β* (f) and TNF-*α* (g) proteins secreted into the cell culture media were measured by ELISA. Data shows all the values from independent samples of *n* = 3, ^*^ *P* < 0.05 versus nonstimulated controls or cells without TAK-242 treatment.

To investigate whether TLR4 associated with IL-1*β* and TNF-*α* expression induced by LPS, we pre-treated SFs with TAK-242 (1 *μ*M) for 2 h prior to LPS exposure. As shown in Figures 5(b), and 5(c), Western blot analyses revealed that treatment of SFs with LPS increased expression of pro-IL-1*β* and TNF-*α*. To provide the further evidence, the specific TLR4 inhibitor, TAK-242 was used to incubate SFs with LPS stimulation. Increased expression of pro-IL-1*β* and TNF-*α* induced by LPS treatment was entirely inhibited by TAK-242 (Figures 5(b), and 5(c)). The same results were also found in their mRNA expression (Figures 5(d), and 5(e)). As shown in Figures 5(f), and 5(g), treatment with TAK-242 significantly reduced LPS-enhanced mature IL-1*β* and TNF-*α* protein levels in the cell culture media. These results suggested that TLR4 involved in expression of IL-1*β* and TNF-*α* induced by LPS.

### 3.5. Effect of MyD88 on the Expression of IL-1*β* and TNF-*α* in SFs

**Figure 6:**
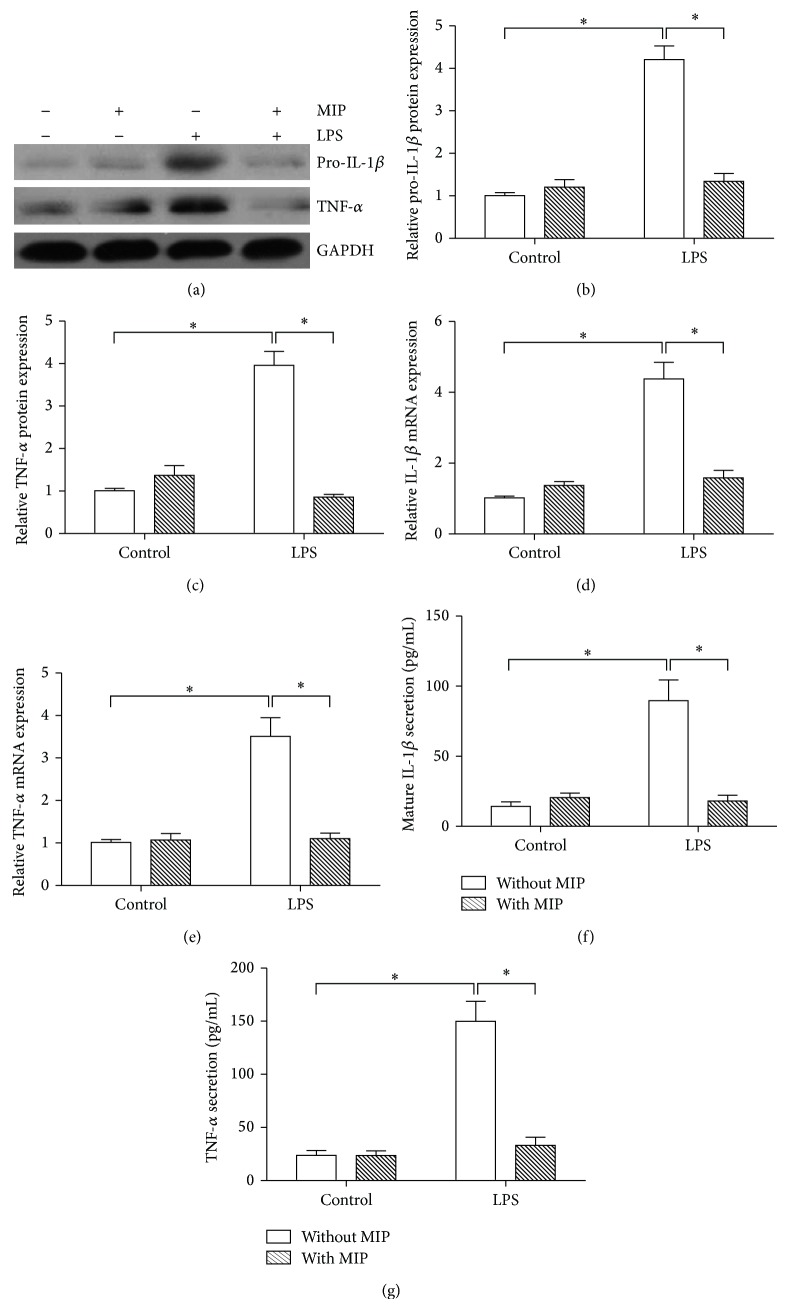
Effect of MyD88 on the expression of IL-1*β* and TNF-*α* in SFs. SFs were pre-treatment with or without MIP, then stimulated with LPS. Cell lysates were collected (at 12 h) for analyzing the protein expression by Western blot (a), and the relative expression of pro-IL-1*β* (b) and TNF-*α* (c) was calculated. Total RNAs were extracted (at 6 h) for detecting mRNAs of IL-1*β* (d) and TNF-*α* (e) by real-time quantitative PCR. The cell-free culture medium from each sample was collected at 12 h, and the mature IL-1*β* (f) and TNF-*α* (g) proteins secreted into the cell culture media were measured by ELISA. Data shows all the values from independent samples of *n* = 3, ^*^ *P* < 0.05 versus nonstimulated controls or cells without MIP treatment.

To confirm the relationship between TLR4 and its downstream molecules MyD88 and the involvement of MyD88 in the effects of LPS in SFs, we pre-treated SFs with MIP (100 *μ*M) for 2 h prior to LPS exposure. The results showed that treatment with MIP could significantly inhibit increased expression of pro-IL-1*β* and TNF-*α* induced by LPS at both protein (Figures 6(b), and 6(c)) and mRNA (Figures 6(d), and 6(e)) levels. Also, treatment with MIP significantly reduced LPS-enhanced mature IL-1*β* and TNF-*α* protein levels secreted to the cell culture media. We speculate that MyD88 is an indispensable adoptor protein in the pathways of intracellular signaling transduction triggered by TLR4 in SFs.

## 4. Discussion

The TMJ synovial membrane lines all of the intra-articular structures, except for articular cartilage of the eminence, fossa and mandibular condyle, and the articular disc. The synovial membrane can secrete synovial fluid into joint cavity, and the fluid has rheologic and ncviscoelastic properties serves lubricational and nutritional function to the joint. Gynther et al. have demonstrated the occurrence of inflammatory reactions in the synovial membrane of TMD patients by arthroscopic and histopathological studies [26]. Wang et al. induced TMJ Osteoarthritis in rats by CFA injection, and found that the TMJ synovium shows features characteristic of chronic synovitis, including proliferation of synovial lining cells, extensive infiltration of mononucleated, putative inflammatory cells in the subsynovial tissue, proliferative and dilated blood vessels and abundant lipid droplets [27]. These studies showed us the important position of synovium in the pathogenesis of TMD. Therefore, synovial tissues were obtained from human and animal, and the SFs of TMJ was cultured in vitro intended for scientific research. The levels of proinflammatory like IL-6 and sIL-6R in SFs were increased with IL-1*β* stimulation, and excessive production of IL-6 seems to be related to abnormalities associated with TMD [28]. Hydrostatic pressures and TNF-*α* stimulation induced increase of cadherin-11, vascular endothelial growth factor (VEGF) and fibroblast growth factor (FGF), and these molecules are known to play a significant role in the formation of inflammation [29]. In the present study, we detected the change of TLR4, MyD88, IL-1*β* and TNF-*α* expression in SFs from TMJ exposed to LPS, an outer membrane component of Gram negative bacteria cell walls [30], Which is a ligand and potent agonist of TLR4. Consistent with these findings, the mRNA and protein expression of proIL-1*β* and TNF-*α* in SFs treated with LPS was evaluated to be positively related to the concentrations and duration time of LPS. Also, LPS increased TNF-*α* and mature IL-1*β* protein levels secreted to the cell culture media in a time-dependent and dose-dependent manner. These researches all support the hypothesis that the SFs is another inflammatory effector cell which participate in innate immune response and inflammation of joint except macrophage, dendritic cell, T-cell.

IL-1*β* is a proinflammatory cytokine that mediates a variety of host defense processes, such as inflammation and cellular response to injury. Its importance in joint destruction appears to result from its ability to suppress the synthesis of type II collagen characteristic of articular cartilage and promote the synthesis of type I collagen characteristic of fibroblasts, induce the production of iNOS, receptor activator of NF-*κ*B ligand and MMPs that induce matrix degradation, and suppress the ability of chondrocytes to synthesize new proteoglycan [31, 32]. Although it was appeared that TNF-*α* act with a potency approximately 10 times lower than that of IL-1*β*, TNF-*α* stimulates the induction of IL-1*β*, and increases the effect of IL-1*β*, coordinately [33]. However, there is still no clear evidence of the intracellular signalling pathways that are responsible for enhanced cytokines expression in TMD. In order to investigate whether all of IL-1*β*, TNF-*α* expression can be upregulated by TLR4- triggered signaling transduction, we pretreat SFs with a TLR4 specific inhibitor TAK-242 before stimulating cells with LPS. The results showed that LPS could increase the expression of IL-1*β* and TNF-*α* at both mRNA and protein levels compared with the control group. However, the effect of LPS was significantly decreased by the use of TAK-242, which could selectively suppress TLR4-mediated MyD88-dependent pathway as well as TRIF-dependent pathway by binding to Cys747 in the intracellular domain of TLR4 and its inhibitory effect is largely unaffected by LPS concentration and types of TLR4 ligands [34, 35]. Our data demonstrated that TAK-242 could significantly inhibit LPS-enhanced IL-1*β* and TNF-*α* expression at both mRNA and protein levels in SFs. These may represent an important link between activation of TLR4 and the increased expression of proinflammatory cytokines like IL-1*β* and TNF-*α*. As we all know, the inflammatory response that involved in TMD is not caused by bacterial infection. LPS is a key component of Gram negative bacteria cell walls and act as exogenous activation of TLR4. In the present study, we only demonstrated that the SFs obtained from TMJ in rats has potential capacity to mediate the expression of proinflammatory cytokines like IL-1*β* and TNF-*α* via the activation of TLR4 signaling. However, little is known about whether the expression of TLR4 increases in synovial lining cell layer of TMJ in patients with TMD and whether there are endogenous ligands in the progression of TMD. We will conduct further researches to explain these questions.

Additionally, our study confirmed the relationship between TLR4 and its downstream molecules MyD88 and the involvement of MyD88 in the effects of LPS in SFs by using MIP, a MyD88 specific inhibitor, which inhibits MyD88 function while not altering its expression [36]. The results showed that treatment with MIP could significantly inhibit LPS-induced IL-1*β* and TNF-*α* protein expression in SFs. We speculate that MyD88 is an indispensable adaptor protein in the pathways of intracellular signaling transduction triggered by TLR4 in SFs. However, whether the other three adaptor molecules TIRAP, TRIF, TRAM participate in intracellular signaling and induce production of inflammatory mediators in SFs is not detected. More studies are necessary to determine whether this is indeed the case.

In summary, the results show that TLR4 is involved in the expression of IL-1*β* and TNF-*α* in SFs from TMJ with LPS stimulation, and the effects are dependent at least in part upon MyD88. Maybe TLR4/MyD88 signal transduction pathway participate in enhanced expression IL-1*β* and TNF-*α* in patients with TMD. The activation of TLR4/MyD88 signal transduction pathway which results in production of proinflammatory factors may play a role in the pathogenesis of TMD. Medicines able to block the TLR4 signal transduction pathway or interfere with ligands binding to the receptor may ameliorate the proinflammatory phenotype and prevent the development of TMD.
